# 影响儿童及青少年慢性髓性白血病患者停用伊马替尼后无治疗缓解结局的相关因素分析

**DOI:** 10.3760/cma.j.cn121090-20250222-00087

**Published:** 2025-09

**Authors:** 慧芳 赵, 倩 江, 纬明 黎, 雨 朱, 兵城 刘, 庆曙 曾, 树霞 郭, 立新 梁, 春蕾 张, 璎玲 祖, 永平 宋, 䶮莉 张

**Affiliations:** 1 郑州大学附属肿瘤医院，河南省肿瘤医院，郑州 450008 Affiliated Cancer Hospital of Zhengzhou University, Henan Cancer Hospital, Zhengzhou 450008, China; 2 北京大学人民医院、北京大学血液病研究所、国家血液系统疾病临床医学研究中心，北京 100044 People's Hospital of Peking University, Peking University Institute of Hematology, National Clinical Medical Research Center for Blood System Diseases, Beijing 100044, China; 3 华中科技大学同济医学院附属协和医院，武汉 436022 Union Hospital, Tongji Medical College, Huazhong University of Science and Technology, Wuhan 436022, China; 4 南京医科大学第一附属医院，江苏省人民医院，南京 210029 The First Affiliated Hospital of Nanjing Medical University, Jiangsu Provincial People's Hospital, Nanjing 210029, China; 5 中国医学科学院血液病医院（中国医学科学院血液学研究所），天津 300020 Institute of Hematology and Blood Diseases Hospital, National Clinical Research Center for Blood Diseases, Chinese Academy of Medical Sciences and Peking Union Medical College Hematology, Tianjin 300020, China; 6 安徽医科大学第一附属医院，合肥 230022 The First Affiliated Hospital of Anhui Medical University, Hefei 230022, China; 7 郑州市人民医院，郑州 450003 Zhengzhou People's Hospital, Zhengzhou 450003, China; 8 三门峡市中心医院，三门峡 472000 Sanmenxia Central Hospital, Sanmenxia 472000, China

**Keywords:** 白血病，髓样，慢性, 儿童及青少年, 伊马替尼, 无治疗缓解, Leukemia, myeloid, chronic, Children and adolescent, Imatinib, Treatment-free remission

## Abstract

**目的:**

分析影响儿童及青少年慢性髓性白血病（CML）患者停用伊马替尼（IM）后无治疗缓解（TFR）结局的相关因素。

**方法:**

回顾性纳入2016年12月1日至2024年9月27日国内8家血液中心停用IM且具有明确停药结局的36例儿童及青少年CML患者，分析其临床特征及停药后治疗反应的演变。使用单因素分析和多因素Cox比例风险回归模型评估影响停用IM治疗CML患者TFR结局的相关因素。

**结果:**

36例患者，男17例，女19例。确诊CML时和停用IM时中位年龄分别为11（5，16）岁和20（14，25）岁。IM治疗至首次获得深层分子学反应（Deep molecular response，DMR）的中位时间21（13，38）个月。停IM前，IM中位治疗96（84，121）个月，DMR持续时间74（63，89）个月。停用IM后中位随访38（15，68）个月，6、12、24、36个月时的累积TFR率分别为74.1％、60.7％、60.7％、56.0％，总体的TFR率为58.3％。15例患者在中位停IM治疗5（3,11）个月失去主要分子学反应（MMR）。15例失去MMR患者全部重启酪氨酸激酶抑制剂治疗，13例重启IM治疗，2例重启达沙替尼治疗。至随访结束，13例（86.7％）患者在中位治疗5（3,17）个月重获DMR，至末次随访无患者出现疾病进展。2例（5.6％）患者发生停药综合征。单因素分析结果显示：停IM前IM治疗时间≥100个月组患者的TFR率较<100个月组显著升高（82.4％对36.8％，*P*＝0.017），停IM前DMR持续时间≥72个月组患者的TFR率较<72个月组显著升高（84.2％对29.4％，*P*＝0.003）。多因素分析结果显示：停药前DMR持续时间是影响儿童及青少年CML患者停IM治疗后TFR结局的独立影响因素（*HR*＝5.419，95％*CI*：1.524～19.272，*P*＝0.009）。

**结论:**

DMR持续时间显著影响儿童及青少年CML患者停IM治疗后TFR结局。停IM前维持DMR≥72个月者TFR率显著提高。

## Abstract (en)

**Objective:**

To identify factors influencing treatment-free remission（TFR）outcomes in children and adolescent patients with chronic myeloid leukemia（CML）after imatinib（IM）discontinuation.

**Methods:**

This multicenter retrospective study analyzed 36 children and adolescent patients with CML from eight hematology centers in China（December 1, 2016, to September 27, 2024）who discontinued IM therapy with documented post-cessation outcomes. Clinical characteristics and molecular response dynamics were assessed. Univariate analysis and multivariate Cox proportional hazards regression models were employed to assess factors associated with TFR outcomes.

**Results:**

A total of 36 patients were documented, comprising 17 males and 19 females. The median ages at CML diagnosis and IM discontinuation were 11 years（*IQR*: 5,16）and 20 years（*IQR*: 14,25）, respectively. The median time from IM initiation to first deep molecular response（DMR）was 21 months（*IQR*: 13, 38）. Pre-discontinuation, patients received IM for a median duration of 96 months（*IQR*: 84, 121）and maintained DMR for 74 months（*IQR*: 63, 89）. With a median post-discontinuation follow-up of 38 months（*IQR*: 15, 68）, cumulative TFR rates at 6, 12, 24, and 36 months were 74.1％, 60.7％, 60.7％, and 56.0％, respectively, generating an overall TFR rate of 58.3％. Fifteen patients lost major molecular response at a median of 5 months post-discontinuation（*IQR*: 3, 11）. All 15 patients resumed tyrosine kinase inhibitor therapy, comprising 13 who restarted IM and 2 who switched to dasatinib. By the last follow-up, 13（86.7％）patients regained DMR after a median treatment duration of 5 months（*IQR*: 3, 17）, and no disease progression occurred in any patient. Withdrawal syndrome occurred in 2（5.6％）patients. Univariate analysis revealed significantly higher TFR rates in patients with pre-discontinuation IM duration of ≥100 months *vs* <100 months（82.4％ *vs 3*6.8％, *P*＝0.017）and pre-discontinuation DMR duration of ≥72 months *vs* <72 months（84.2％ *vs* 29.4％, *P*＝0.003）. Multivariate Cox analysis identified pre-discontinuation DMR duration as an independent protective factor for TFR（*HR*＝5.419, 95％*CI*: 1.524–19.272, *P*＝0.009）.

**Conclusion:**

DMR duration was identified as an independent protective factor influencing TFR outcomes in children and adolescent patients with CML after IM discontinuation. Patients who maintained DMR for ≥72 months before IM discontinuation demonstrated a significantly higher TFR rate.

酪氨酸激酶抑制剂（TKI）时代，儿童及青少年慢性髓性白血病（CML）患者的预期寿命同正常同龄人群接近[1]–[3]。提高生活质量和实现无治疗缓解（Treatment-free remission, TFR）逐渐成为儿童及青少年CML治疗追求的新目标。儿童CML发病率低，占儿童白血病的2％～3％[4]。目前已发表的停药研究多为小样本回顾性研究，且主要聚焦于伊马替尼（IM）[5]–[9]。儿童及青少年CML较成人CML更具侵袭性，盲目停药可能导致较高复发风险。因此，探索影响儿童及青少年CML TFR结局的因素显得尤为重要。本研究回顾性收集来自国内8家血液病治疗中心的36例停用IM的CML患者临床资料，系统探讨影响TFR结局的关键因素，旨在为儿童及青少年CML患者安全停用IM提供循证依据。

## 病例与方法

1. 病例：本研究共纳入自2016年12月1日至2024年9月27日期间停止IM治疗的确诊时年龄<18岁且经IM一线治疗获得深层分子学反应（Deep molecular response，DMR）≥2年的36例儿童及青少年CML慢性期（CP）患者。所有患者的诊断符合文献[10]标准。36例患者分别来自河南省肿瘤医院、北京大学人民医院、华中科技大学同济医学院附属协和医院、南京医科大学第一附属医院、中国医学科学院血液病医院、安徽医科大学第一附属医院、郑州市人民医院、三门峡市中心医院等国内8家血液病治疗中心。

入组标准：①确诊CML时年龄<18岁的儿童及青少年CML-CP患者；②IM一线治疗且在规范国际标准检测下维持稳定DMR≥2年；③有强烈停IM意愿和（或）发生药物相关不良反应以及因其他因素被迫停药。排除标准：①转录本非BCR::ABL210融合基因阳性患者；②既往接受过造血干细胞移植的患者。本研究已通过河南省肿瘤医院伦理委员会批准（2016096），并豁免知情同意。

收集患者的人口学资料、Sokal评分、ELTS评分、停药前IM的治疗时间、治疗反应、DMR持续时间、停药的原因，以及停IM后BCR::ABL融合基因的变化情况、患者症状的变化等临床资料。

2. 疗效评估标准、定义和停药后疾病监测频率：疗效评估标准参照《慢性髓性白血病中国诊断与治疗指南（2020年版）》[10]。主要分子学反应（MMR）的定义为BCR::ABL融合基因^IS^≤0.1％（ABL转录本>10 000）；分子学反应4.0（Molecular response 4.0，MR4.0）的定义为BCR::ABL融合基因^IS^≤0.01％（ABL转录本>10 000），分子学反应4.5（Molecular response 4.5，MR 4.5）的定义为BCR::ABL融合基因^IS^≤0.003 2％（ABL转录本>32 000）；DMR的定义为分子学水平在MR 4.0或MR 4.5（实时荧光定量PCR检测敏感度≥MR 4.5）。

停药后分子学监测频率：前6个月每1～2个月1次，第6～12个月每2～3个月1次，12个月后每3～6个月1次。失去MMR后，建议尽快重启TKI治疗。

TFR时间指接受IM治疗获得持续稳定DMR≥2年后，从首次停药开始至失去MMR或因任何原因重启TKI治疗的间隔时间。停药综合征指停用IM后数周至数月内出现的以肌肉、骨骼疼痛为特征的临床症状群。

3. 随访：通过查看门诊记录及电话联系的方式随访。自停IM开始随访，随访截止时间为2024年12月11日。

4. 统计学处理：采用IBM SPSS 25.0和GraphPad Prism 8.0软件分别进行统计学分析与图表绘制。患者人口学及临床特征采用描述性分析方法。偏态分布计量资料采用中位数（四分位距）［*M*（*IQR*）］表示。定量资料比较采用Mann-Whitney *U*检验，分类资料比较采用*χ*²检验或Fisher确切概率法。采用受试者工作特征（ROC）曲线确定确诊年龄、停药年龄、IM治疗至获DMR时间、停药前IM治疗时间、DMR持续时间等可能影响患者TFR结局的连续变量的最佳截断值。其中确诊年龄最佳截断值为11岁、停药年龄为20岁、IM治疗至获DMR时间为34个月、停药前IM治疗时间为100个月、DMR持续时间为72个月，以最佳截断值为界分为两组。应用Kaplan-Meier法分析事件累积发生率，组间TFR率差异采用Log-rank检验进行单因素分析，多因素分析采用Cox比例风险回归模型。以*P*<0.05为差异有统计学意义。

## 结果

1. 基线特征：36例儿童及青少年CML患者确诊时染色体均为典型Ph染色体。停药前，36例患者中，19例（52.7％）IM治疗时间≥96个月，28例（77.1％）≥84个月，31例（86.1％）≥72个月，35例（97.2％）≥60个月，仅1例IM治疗59个月；19例（52.7％）患者DMR持续时间≥72个月，28例（77.7％）≥60个月，34例（94.4％）≥48个月。36例停用IM治疗的儿童及青少年CML患者的基线特征见表1。停药原因包括：疗效持续DMR在医师指导下停药者14例（38.9％），自行停IM者12例（33.3％），女性患者进入育龄期后因意外妊娠停IM者4例（11.1％），因生长发育迟缓停IM者4例（11.1％），因子宫肌瘤手术停IM者1例（2.8％），因新型冠状病毒肺炎疫情无法购药被迫停IM者1例（2.8％）。

**表1:** 36例停用IM治疗的儿童及青少年CML患者基线特征

特征	数值
确诊CML时年龄［岁，*M*（*IQR*）］	11（5，16）
停TKI时年龄［岁，*M*（*IQR*）］	20（14，25）
性别（例，男/女）	17/19
Sokal评分［例（％）］	
低危	21（58.3）
中高危	7（19.4）
未知	8（22.2）
ELTS评分［例（％）］	
低危	23（63.9）
中高危	5（13.9）
未知	8（22.2）
IM治疗至获得MMR时间［月，*M*（*IQR*）］	7（3，13）
IM治疗至获得DMR时间［月，*M*（*IQR*）］	21（13，38）
停IM前IM治疗时间［月，*M*（*IQR*）］	96（84，121）
停IM前DMR持续时间［月，*M*（*IQR*）］	74（63，89）
随访时间［月，*M*（*IQR*）］	38（15，68）

**注** IM：伊马替尼；CML：慢性髓性白血病；TKI：酪氨酸激酶抑制剂；MMR：主要分子学反应；DMR：深层分子学反应

2. 停用IM后患者的TFR结局：36例患者停IM后，中位随访38（15，68）个月，6、12、24、36个月的TFR率分别为74.1％、60.7％、60.7％、56.0％，总体的累积TFR率为58.3％。36例停IM治疗的儿童及青少年CML患者的累积TFR曲线见图1。

**图1:**
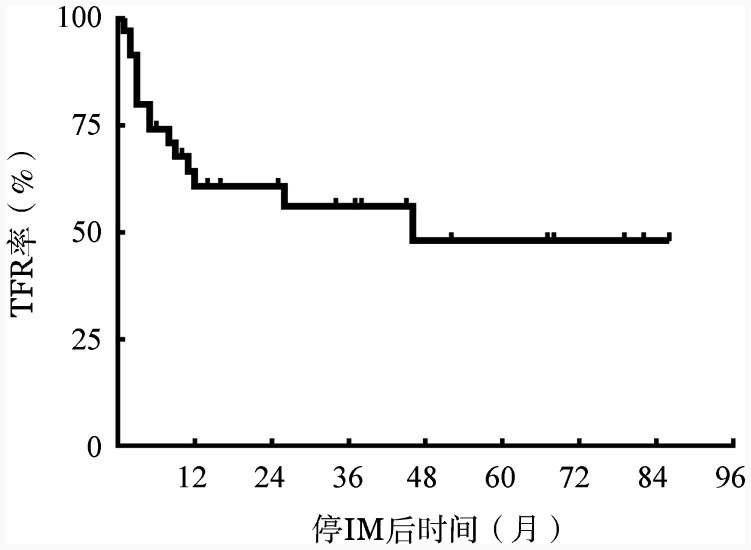
36例停用伊马替尼（IM）治疗的儿童及青少年慢性髓性白血病（CML）患者累积无治疗缓解（TFR）曲线

3. 停用IM后失去MMR患者重启TKI治疗情况以及疗效转归：15例（41.7％）患者在中位停IM治疗5（3，11）个月后失去MMR，在确认失去MMR后均立即重启TKI治疗，其中13例重启IM治疗，2例重启达沙替尼治疗。至随访结束，13例（86.7％）患者在重启TKI中位治疗5（3，17）个月重获DMR。15例失去MMR后重启TKI治疗患者的累积DMR曲线见图2。另外2例患者，1例重启治疗后随访时间尚短，另1例重启IM治疗19个月BCR::ABL^IS^ 0.16％。15例患者无一例在失去MMR后出现疾病进展。2例患者发生停药综合征，表现为轻度骨痛，口服解热镇痛药物可缓解。

**图2:**
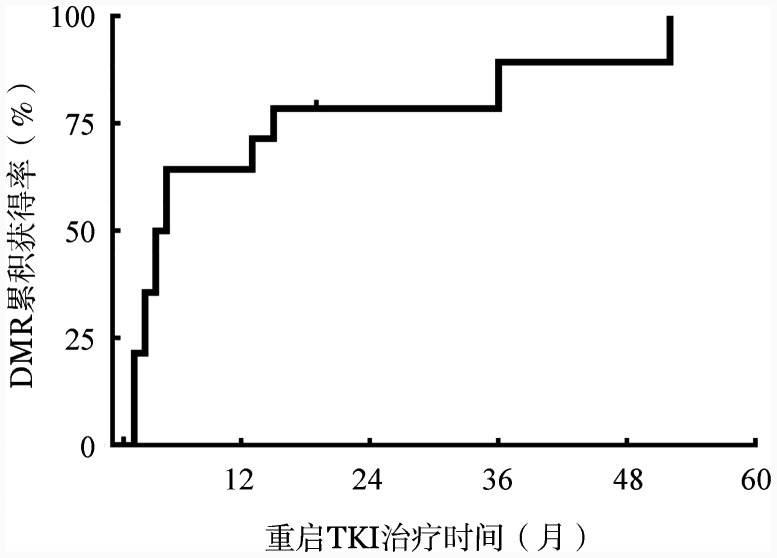
15例失去主要分子学反应后重启酪氨酸激酶抑制剂（TKI）治疗患者的累积深层分子学反应（DMR）曲线

4. 影响儿童及青少年CML停IM治疗后TFR结局的单因素和多因素分析：将性别、年龄、Sokal评分、ELTS评分、IM治疗至获DMR时间、停药前IM治疗时间、DMR持续时间等变量纳入单因素分析，结果显示：停药前IM治疗时间≥100个月组患者的TFR率较<100个月组显著升高（82.4％对36.8％，*P*＝0.017）（图3）；停IM前DMR持续时间≥72个月组患者的TFR率较<72个月组显著升高（84.2％对29.4％，*P*＝0.003）（图4）。未发现年龄、性别、Sokal评分、ELTS评分对儿童及青少年CML患者停IM后的TFR结局存在影响（*P*值均>0.05）。单因素分析结果详见表2。将*P*<0.05的变量纳入多因素Cox风险比例模型进行分析，结果显示：停药前DMR持续时间是影响TFR结局的独立因素（*HR*＝5.419,95％*CI*：1.524～19.272，*P*＝0.009）。

**图3:**
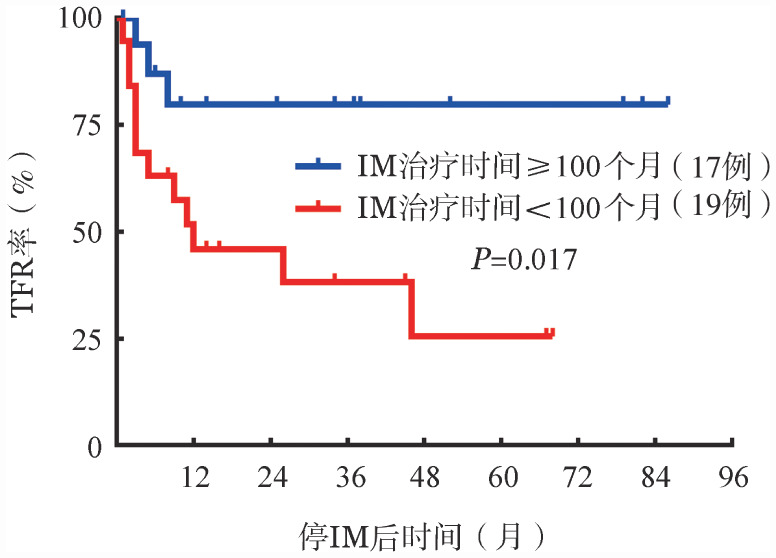
停药前伊马替尼（IM）治疗时间不同组患者的累积无治疗缓解（TFR）曲线

**图4:**
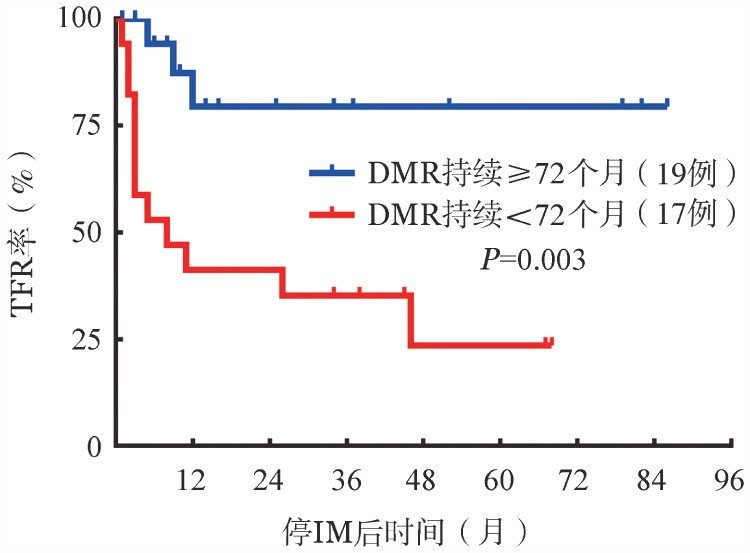
停药前深层分子学反应（DMR）持续不同时间组患者的累积无治疗缓解（TFR）曲线

**表2:** 影响儿童及青少年慢性髓性白血病患者停用IM后TFR结局的单因素分析结果

特征	例数	TFR率（％）	*χ*^2^值	*P*值
性别			0.165	0.685
男	17	58.8		
女	19	57.9		
确诊时年龄			0.358	0.550
≥11岁	18	50.0		
<11岁	18	66.7		
停IM时年龄			0.699	0.403
≥20岁	7	42.9		
<20岁	23	60.9		
Sokal评分			1.028	0.311
低危	21	57.1		
中高危	7	28.6		
ELTS评分			0.586	0.444
低危	23	52.2		
中高危	5	40.0		
IM治疗至获得DMR时间			2.520	0.112
≥34个月	10	40.0		
<34个月	26	65.4		
停药前IM治疗时间			5.697	0.017
≥100个月	17	82.4		
<100个月	19	36.8		
停药前DMR持续时间			8.870	0.003
≥72个月	19	84.2		
<72个月	17	29.4		

**注** IM：伊马替尼；TFR：无治疗缓解；DMR：深层分子学反应

## 讨论

TKI长期治疗在为儿童及青少年CML患者带来生存获益的同时，其相关脱靶效应也给处于特殊生长发育阶段的患者带来独特挑战。青春期前患者要面临TKI相关生长发育迟缓的风险，青春期患者需关注性激素水平异常及甲状腺功能紊乱等内分泌毒性，以及育龄期女性患者的卵巢储备功能下降及潜在生育力受损等问题，均严重影响儿童及青少年CML的生活质量[1]–[3]。本研究中患者的主要停药原因包括：疗效持续DMR在医师指导下停药（38.9％）、自行停药（33.3％）、女性患者进入育龄期后因意外妊娠被迫停药（11.1％）、因生长发育迟缓停药（11.1％）、因子宫肌瘤手术停药（2.8％）、因新型冠状病毒肺炎疫情无法购药被迫停药（2.8％）等。由此可见，追求TFR同样是儿童及青少年CML期待实现的远期目标之一。

研究显示，40％～60％TKI治疗获得持续DMR≥2年的成人CML患者，停药后可获得TFR[11]–[14]。而符合成人停药标准的儿童及青少年CML患者的TFR率却存在明显差异（28.6％～72％）[5],[7]–[8],[15]–[16]。STOP IMAPED研究将失去MR4.0定义为分子学复发，14例患儿停药前接受IM中位治疗64个月，DMR中位持续49个月，6个月TFR率28.6％[5]。而在另一项采用同样严格分子学复发标准（检测到BCR::ABL1转录本定义为分子学复发）的儿童停药研究中，11例IM治疗≥5年且持续分子学阴性≥2年的患儿，TFR率高达72.7％（8/11）[16]。而将“失去MMR”作为分子学复发标准后，尽管儿童及青少年CML患者的TFR率有所提高，但不同研究中相同停药时间节点的TFR率差异仍较为明显。儿童CML国际注册中心研究数据[7]显示，18例患儿，停药前IM中位治疗73.2个月，MR4.0中位持续46.2个月，6、12、36个月的TFR率为61％、56％、56％。来自日本的儿童及青少年停药研究（JPLSG STKI-14研究）结果显示，停药前TKI中位治疗时间100个月，MR4.0的中位持续时间为53.5个月，12个月的TFR为50.0％[8]。近期一项来自印度的前瞻性、单中心研究[15]结果显示，54例患儿停药前IM中位治疗126个月，DMR中位持续54个月，12个月和24个月TFR率为70％和66％。本研究数据显示，36例患儿停药前IM中位治疗96个月、DMR中位持续74个月，停药后6、12、24、36个月的TFR率依次为74.1％、60.7％、60.7％、56.0％。综上可见，部分符合成人停药标准的儿童及青少年CML患者停IM后可以实现TFR。但需注意：儿童及青少年CML较成人CML具有更强的疾病侵袭性，且现有数据显示该群体停用IM后TFR率存在显著异质性。因此，非常有必要进一步探索影响停用IM儿童及青少年CML患者TFR的因素。

目前儿童及青少年CML患者停药后TFR结局的影响因素尚未明确。本研究单因素分析显示，IM治疗时间≥100个月组TFR率较<100个月组显著升高，停IM前DMR持续时间≥72个月组TFR率较<72个月组显著升高。多因素分析证实停药前DMR持续时间是TFR的独立保护因素（*HR*＝0.107，*P*＝0.004）。值得注意的是，JPLSG STKI-14研究[8]和Moulik等[15]的研究虽纳入相同变量，但未发现IM治疗时间和DMR持续时间对TFR的影响，可能与本研究队列患者停药前DMR中位持续时间（74个月）较既往研究报道（49、46.2、53.5、54个月）[5],[7]–[8],[15]更长有关。而DMR持续时间对于成人CML患者TFR结局的预后价值已被多项研究证实。EURO-SKI研究[11]显示，更长的IM治疗持续时间和更长的DMR持续时间与6个月的MMR维持率显著相关。一项纳入23项停药研究的Meta分析进一步指出：DMR持续时间、分子学反应深度及干扰素治疗史是影响TFR结局的关键因素[17]。由此可见，相较于IM治疗时间，更长的DMR持续时间对改善儿童及青少年CML患者的TFR结局可能具有更显著的临床价值。

综上，本研究结果显示DMR持续时间是影响接受IM治疗的儿童及青少年CML患者TFR结局的独立因素，当DMR持续时间≥72个月时，患者获得TFR的成功率显著提高，这为临床实践中制定儿童及青少年CML患者TFR策略提供了循证依据。但本研究尚存在以下局限性：①为回顾性研究；②样本量较小。其结论后续需通过前瞻性、多中心、大样本临床研究进一步验证。
